# Diagnostic value of a serum four-biomarker panel for atrophic gastritis in women under 60 years of age

**DOI:** 10.3389/fmed.2026.1945445

**Published:** 2026-09-25

**Authors:** Wenqiao Jia, Wei Han

**Affiliations:** Department of Health Management Center, Qilu Hospital of Shandong University, Jinan, China

**Keywords:** atrophic gastritis, gastric function triple-parameter, gastrin-17 (G-17), pepsinogen I, pepsinogen II

## Abstract

**Background:**

While endoscopy is considered the gold standard for diagnosing chronic atrophic gastritis (CAG), serum biomarkers such as pepsinogen I (PG1), pepsinogen Ⅱ (PG2), the PGⅠ/PGⅡ ratio, and gastrin-17 (G-17) have been investigated for their diagnostic value in CAG. However, studies to date have reported inconsistent results regarding their accuracy and clinical utility.

**Methods:**

All 567 participants underwent upper gastrointestinal endoscopy and serum biomarkers were examined for PG1, PG2 and G-17 using Gastric Function Triple-Parameter test kit (Biohan Biotechnology Co., Ltd). The general characteristics of the participants were derived from the physical examination data from Qilu Hospital of Shandong University.

**Results:**

Age was identified as a risk factor of CAG (*p* = 0.01). By incorporating sex and age as covariates into the ROC curve analysis, The serum gastric function biomarkers could serve as diagnostic tools for AG, particularly among female under 60 years old. Within this subgroup, a PG2 cut-off value of ≥6.145 μg/L (AUC = 0.807, sensitivity = 0.762, specificity = 0.800) and a PG1/PG2 ratio cut-off of ≤20.67 (AUC = 0.806, sensitivity = 0.762, specificity = 0.722) showed the strongest discriminatory performance for diagnosing CAG. Meanwhile, PG1 and the PG1/PG2 ratio were significantly associated with the severity of inflammation (*p* = 0.002 and 0.008, respectively).

**Conclusions:**

Serum gastric function biomarkers could serve as diagnostic tools for AG, particularly among female patients under 60 years old of age. Meanwhile, PG1 and the PG1/PG2 ratio were significantly associated with the severity of inflammation.

## Introduction

Gastric atrophy (GA) and gastric intestinal metaplasia (GIM) together characterize chronic atrophic gastritis (CAG), a premalignant condition that can progress to gastric adenocarcinoma (GC) (1). The most common causes of this premalignant lesion are *Helicobacter pylori* (*H. pylori*) infection and autoimmune gastritis. The loss of functional gastric mucosa leads to an acid-depleted environment, which is hypothesized as an early precursor to distal gastric cancer (1). Endoscopic monitoring is recommended for patients with severe atrophic gastritis (2). Given its chronic inflammatory and hypochlorhydric nature with malignant potential, effective screening for CAG is essential in high-risk populations (3).

Most patients with CAG remain asymptomatic in early stages. Even in advanced stages, symptoms may be non-specific, such as upper gastrointestinal discomfort, manifestations of autoimmune diseases, or pernicious anemia. Although endoscopic biopsy histology remains the gold standard of diagnosis of CAG (4), its use is limited by invasiveness, high cost, and a shortage of skilled endoscopists and equipment. Therefore, there is a critical need to develop non-invasive, highly reliable methods for detecting CAG or stratify high-risk patients.

Serum biomarkers such as pepsinogen Ⅰ (PG1), pepsinogen Ⅱ (PG2), the PG1/PG2 ratio, and gastrin-17 (G-17) have been studied for their diagnostic utility in CAG. However, previous reports have shown conflicting results. Some evidence supports their use in CAG diagnosis, but their accuracy remains controversial. For instance, Yu Haitao et al. demonstrated that combining these 4 serum biomarkers with anti- *H. pylori* antibodies testing enhances CAG screening efficacy (5). In contrast, Cosmin et al. found that the biomarker panel used in GastroPanel lacks sufficient diagnostic accuracy for detecting gastric atrophy in their studied population, with the PG1/PG2 ratio showing particularly low reliability in patients with multifocal atrophy (6). Sergey et al. also recommended postprandial G-17 for dynamic monitoring of atrophic gastritis after treatment (7). Valli et al. demonstrated pepsinogens to distinguish patients with gastric intestinal metaplasia and *H. pylori* infection among populations at risk for GC (8).

Although biomarkers have been widely studied, the relationship between serum gastric function biomarkers and CAG remains unclear. Meanwhile, few investigations have focused on women under 60 years of age, a subgroup in which diagnostic performance may differ. Our study is the first to systematically evaluate a four-biomarker panel in this population. In this study, we systematically analyzed it and we identified PG2 and PG1/PG2 could serve as diagnostic biomarkers for AG, especially in female patients with age under 60 years old. Meanwhile, PG1 and PG1/PG2 were significantly related with the severity of inflammation.

### Patients, methods and material

The study enrolled 567 participants who underwent the health management in Qilu Hospital of Shandong University during 2021 January to 2023 July (Figure 1). Upper gastrointestinal endoscopy was performed by gastroenterologists to all participants and pathological examinations of biopsy samples were conducted by one single expert pathologist. This was a retrospective study, and the number and sites of biopsy samples were determined by the operating endoscopists according to routine clinical practice, rather than following a standardized biopsy protocol. 77 participants showed histological mucosal atrophy. All 567 participants underwent endoscopic method were examined for PG1, PG2 and G-17 using Gastric Function Triple-Parameter test kit (Biohan Biotechnology Co., Ltd). The test results were interpreted according to the reference ranges provided by the kit manufacturer. The normal reference ranges were defined as follows: PG1 >70 μg/L, PG 20–11 μg/L, G-17 0–5.7 pmol/L, and a PG1/PG2 ratio of >7 was considered normal. Serum levels of PG1, PG2, and G-17 were measured based on the fluorescence immunochromatography assay (FICA). The laboratory technicians who performed the assays and the clinicians who collected the samples were double-blinded to the participants' clinical data and test results, respectively.

**Figure 1:**
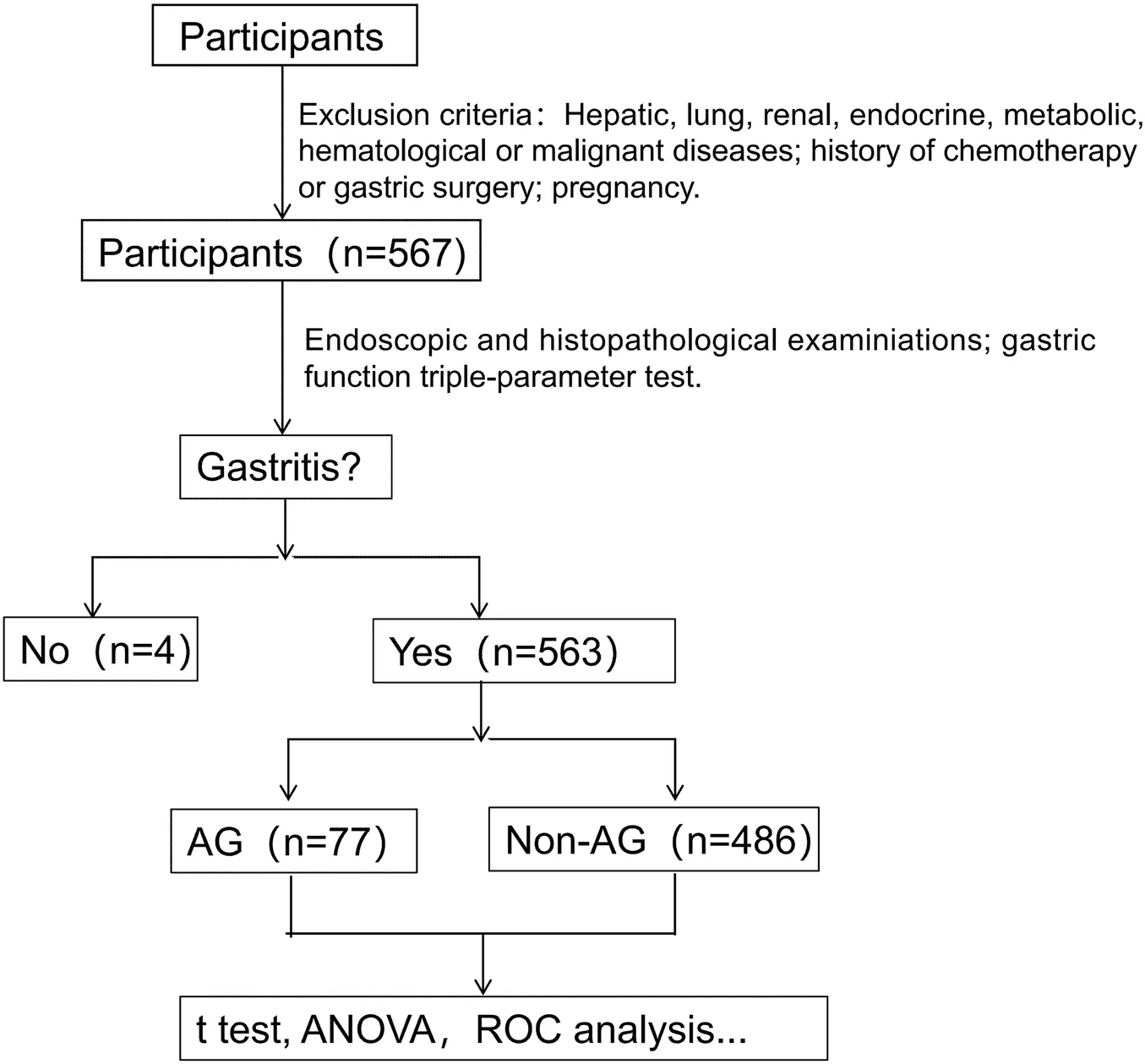
Clinical pathway flowchart for all the participants.

Exclusion criteria were as follows: Hepatic, lung, renal, endocrine, metabolic, hematological or malignant diseases; history of chemotherapy or gastric surgery; pregnancy.

In the present study, the severity of gastric mucosal atrophy was assessed according to the Chinese Consensus on Chronic Gastritis (Chinese Society of Gastroenterology, 2006; updated in 2012 and 2017), which is the standard pathological grading system routinely used in clinical practice in China. According to this system, the degree of atrophy is classified into four grades based on the proportion of reduced native gastric glands: Mild (+): reduction of native glands by <1/3; Moderate (++): reduction by 1/3 to 2/3; Severe (+++): reduction by >2/3 or complete loss.

Ethical approval was obtained from the Medical Ethics Committee of Qilu Hospital of Shandong University (KYLL-202212-032-1). The measurement of serum PG1,PG2 and G-17 were carried out in the Department of Clinical Laboratory.

### Statistical analysis

The Student's t-test was employed to evaluate the normal distribution of the data. Receiver operating characteristic (ROC) curves were generated to determine the optimal cut-off values for the respective groups. Analysis of variance (ANOVA) was performed to examine the association between biomarker levels and the severity of atrophy or inflammation. A *p*-value of less than 0.05 was considered statistically significant.

## Results

### General characteristics of the participants

A total of 567 participants were included in this study. The characteristics of 567 participants were summarized in Table 1. Among them, there were 186 females (32.8%) and 381 (67.2%) males. 440 (77.6%) patients were under 60 years old, 127 (20.6%) were over 60 years old. Additionally, 117 (20.8%) participants were smokers and 243 were drinkers (42.9%). Based on pathological examination of biopsy samples, 4 (0.7%) participants were not diagnosed with gastritis, 486 (85.7%) were diagnosed with non-atrophic gastritis (non-AG) and 77 (13.6%) were diagnosed with AG. Among all participants, 84 (14.8%) were infected with *H. pylori*.

**Table 1:** The general characteristics of the participants .

Characteristics	*N*(%)
Sex	
Female	186 (32.8)
Male	381 (67.2)
Age	
<60	440 (77.6)
≥60	127 (22.4)
Smoking	
Yes	117 (20.6)
No	450 (79.4)
Drinking	
Yes	243 (42.9)
No	324 (57.1)
Gastritis	
No	4 (0.7)
AG	77 (13.6)
Non-AG	486 (85.7)
H. pylori	
Yes	84 (14.8)
No	483 (85.2)

AG, atrophic gastritis; H. pylor, Helicobacter pylori.

### Statistical analysis of biomarkers depending on different characteristics

Statistical analysis of biomarkers depending on different characteristics was showed in Table 2. Significant differences in biomarker values were observed between sexes (PG1, *p* = 0.002; PG1/PG2, *p* = 0.005, respectively). Notable variations were also found across age groups (PG1, *p* < 0.001; PG2, *p* < 0.001; PG1/PG2, *p* = 0.007; G-17, *p* < 0.001, respectively). No significant differences were associated with smoking or drinking habits. Furthermore, serum levels of PG1, PG2, and G-17 increased, while the PG1/PG2 ratio decreased, in participants with *H. pylori* infection (*p* < 0.001, *p* < 0.001, *p* < 0.001, and *p* = 0.002, respectively).

**Table 2:** Statistical analysis of biomarkers depending on different characteristics.

Characteristics	PG1 (Mean ± SD)	*p*	PG2 (Mean ± SD)	*p*	PG1/PG2 (Mean ± SD)	*p*	G-17 (Mean ± SD)	*p*
Sex		**0**.002**		0.607		**0**.005**		0.179
Female	121.71 ± 49.97		6.13 ± 4.41		23.17 ± 8.71		5.94 ± 9.71	
Male	136.64 ± 55.03		5.95 ± 3.60		25.67 ± 10.40		4.81 ± 9.24	
Age		**<0**.001***		**<0**.001***		**0**.007**		**<0**.001***
<60	126.91 ± 47.05		5.58 ± 3.36		25.45 ± 9.90		4.35 ± 7.63	
≥60	148.50 ± 70.29		7.50 ± 5.05		22.760 ± 9.85		8.07 ± 13.54	
Smoking		0.272		0.241		0.209		0.741
Yes	136.61 ± 49.13		5.63 ± 2.25		25.88 ± 8.98		4.93 ± 8.88	
No	130.48 ± 54.98		6.11 ± 4.20		24.58 ± 10.17		5.245 ± 9.54	
Drinking		0.735		0.181		0.212		0.273
Yes	132.63 ± 51.19		5.76 ± 3.68		25.45 ± 8.80		4.68 ± 8.67	
No	131.08 ± 55.81		6.20 ± 4.02		34.40 ± 10.71		5.55 ± 9.91	
*H. pylori*		**<0**.001***		**<0**.001***		**0**.002**		**<0**.001***
Yes	157.81 ± 65.96		7.74 ± 3.57		21.81 ± 7.28		8.34 ± 10.46	
No	127.21 ± 50.15		5.71 ± 3.86		25.38 ± 10.25		4.63 ± 9.11	

PG1, pepsinogen I; PG2, pepsinogen Ⅱ; G-17, gastrin-17; SD, standard deviation; H. pylor, Helicobacter pylori.

Bold values indicate statistical significance (*p* < 0.05).

*p* < 0.05.

** *p* < 0.01.

*** *p* < 0.001.

### Analysis of risk factors of AG

The analysis of risk factors of AG is presented in Table 3. Age was identified as a risk factor of AG (*p* = 0.01). The mean age of AG patients was 56.065 ± 7.001 years, compared to 52.089 ± 10.021 years in non-AG participants. No significant differences were observed between the AG and non-AG groups in terms of sex, smoking, or drinking habits (*p* = 0.298, 0.762, and 0.079, respectively).

**Table 3:** Analysis of risk factors of AG.

Characteristics	Non-AG	AG	*p*
Sex (*n*)			0.298
Female	154	29	
Male	332	48	
Age (years, Mean ± SD)	52.09 ± 10.02	56.065 ± 7.001	**0**.001**
<60	384	53	**0**.046*
≥60	102	24	
Smoking (*n*)			0.762
Yes	102	15	
No	384	62	
Drinking (*n*)			0.079
Yes	216	26	
No	270	51	
PG1 (μg/L, Mean ± SD)	130.07 ± 52.18	143.33 ± 63.41	**0**.045*
PG2 (μg/L, Mean ± SD)	5.54 ± 2.88	8.95 ± 7.00	**<0**.001***
PG1/PG2 (Mean ± SD)	25.63 ± 9.94	20.28 ± 8.75	**<0**.001***
G-17 (pmol/L, Mean ± SD)	4.82 ± 9.09	7.61 ± 11.12	**0**.015*
*H. pylori (n)*			0.058
Yes	67	17	
No	419	60	

AG, atrophic gastritis; H. pylor, Helicobacter pylori; SD, standard deviation.

Bold values indicate statistical significance (*p* < 0.05).

* *p* < 0.05.

** *p* < 0.01.

*** *p* < 0.001.

Interestingly, all serum biomarker values were significantly altered in AG patients compared to non-AG participants. PG1 was significantly elevated in AG group (143.329 ± 63.409 μg/L) relative to non-AG group (130.07 ± 52.18 μg/L) (*p* = 0.045). PG2 was also significantly higher in the AG group (8.953 ± 6.995 μg/L) than in the non-AG group (5.535 ± 2.878 μg/L) (*p* < 0.001). The values of PG1/PG2 was significantly decreased in AG group (20.280 ± 8.748) compared with non-AG group (25.634 ± 9.937) (*p* < 0.001). Additionally, G-17 levels were significantly increased in the AG group (7.612 ± 11.116 μg/L) relative to non-AG group (4.815 ± 9.092 μg/L) (*p* = 0.015). However, no significant difference in *H. pylori* infection was observed between the AG and non-AG groups (*p* = 0.058).

### Diagnostic value of GastroPanel of AG

We performed stratified ROC analyses according to sex and age groups (<60 years vs. ≥60 years). By incorporating sex and age as covariates into the ROC curve analysis, we found the serum gastric function biomarkers could serve as diagnostic tools for AG, particularly among female patients under 60 years old (Figure 2). Within this subgroup, a PG2 cut-off value of ≥6.145 μg/L (AUC = 0.807, sensitivity = 0.762, specificity = 0.800) and a PG1/PG2 ratio cut-off of ≤20.67 (AUC = 0.806, sensitivity = 0.762, specificity = 0.722) showed the strongest discriminatory performance for diagnosing AG. The serum biomarkers did not show particularly notable diagnostic performance in the other subgroups. In males under 60 years of age, PG2, the PG1/PG2 ratio, and G-17 yielded statistically significant results; however, their AUC values indicated poor diagnostic efficacy. The detailed results were as follows: PG2 at a cut-off value of ≥7.645 μg/L (AUC = 0.665, sensitivity = 0.469, specificity = 0.922, *p* = 0.009); PG1/PG2 ratio at a cut-off value of ≤18.706 μg/L (AUC = 0.667, sensitivity = 0.469, specificity = 0.870, *p* = 0.003); and G-17 at a cut-off value of ≥3.640 μg/L (AUC = 0.645, sensitivity = 0.563, specificity = 0.758, *p* = 0.015). In both males and females ≥ 60 years old, none of the four serum biomarkers showed statistically significant results. These findings collectively indicate that the diagnostic value of these markers is largely limited to other subgroups.

**Figure 2:**
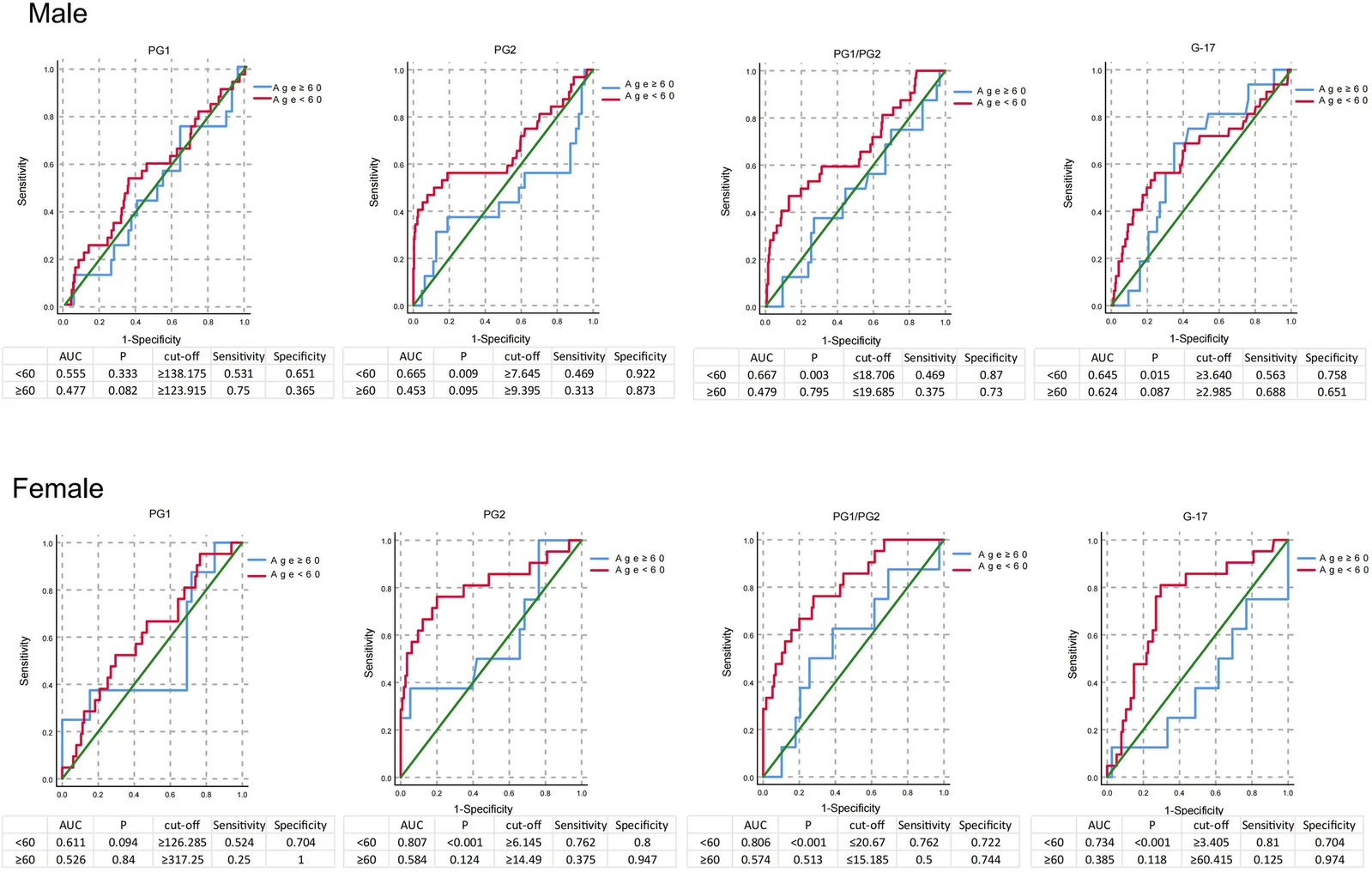
ROC curves of PG1, PG2, PG1/PG2 ratio, G-17, for AG diagnosis across different sex and age subgroups.

Subsequently, the combination of PG2 and PG1/PG2 was used to predict AG under 60 years old (AUC = 0.828, sensitivity = 0.714, specificity = 0.852) (Figure 3). Although sensitivity decreased, the specificity of the combined approach improved compared with individual markers, suggesting that the combined model may help reduce false-positive results in certain clinical scenarios.

**Figure 3:**
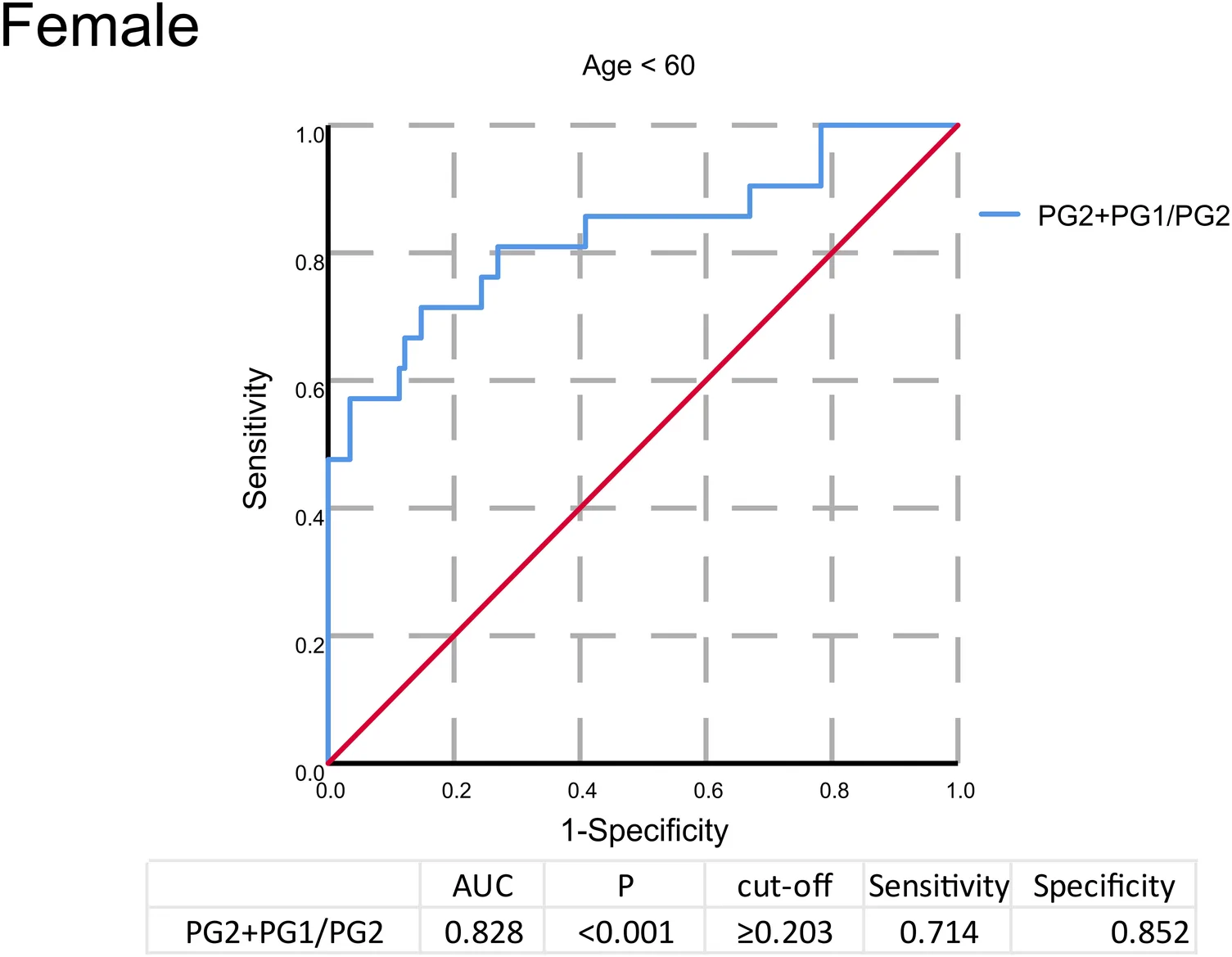
The combination of PG2 and PG1/PG2 ratio for AG diagnosis in female under 60 years old.

Given that *H. pylori* is a core etiological factor in CAG and that our sample size is relatively modest, we fully acknowledge that the observed *p*-value of 0.058 is indeed close to the conventional significance threshold. To further address this concern, we have now performed a stratified subgroup analysis based on *H. pylori* infection status (Supplementary Figures S1, S2). In the *H. pylori*-infected group, both PG2 and the PG1/PG2 ratio demonstrated diagnostic value in individuals aged <60 years, regardless of gender. In the *H. pylori*-negative group, PG2 and the PG1/PG2 ratio showed diagnostic value exclusively in females under 60 years of age.

### The relationship between GastroPanel biomarkers values and the severity of atrophy

The severity of gastric atrophy is associated with the life time risk of developing GC. Previous studies have suggested that PG1, PG2 or G-17 may help evaluate the severity of AG. However, no significant differences were observed in the limited sample of this study (Table 4).

**Table 4:** The relationship between biomarker values and the severity of atrophy.

Atrophy	Mild	Medium	Severe	*p*
PG1 (Mean ± SD)	151.25 ± 71.21	139.95 ± 55.79	127.06 ± 59.11	0.527
PG2 (Mean ± SD)	8.72 ± 5.18	9.75 ± 9.12	7.23 ± 4.43	0.594
PG1/PG2 (Mean ± SD)	20.06 ± 7.80	20.05 ± 8.85	21.81 ± 11.97	0.842
G-17 (Mean ± SD)	7.03 ± 8.82	9.06 ± 14.42	5.03 ± 4.16	0.560

SD, standard deviation.

### The relationship between GastroPanel biomarkers values and severity of inflammation

The relationship between serum gastric fucntion biomarker values and the severity of inflammation is a crucial area of clinical research, with important implications for non-invasive diagnosis, risk stratification, and treatment monitoring. This study found that PG1 and the PG1/PG2 ratio were significantly associated with the severity of inflammation (*p* = 0.002 and 0.008, respectively). In contrast, neither PG2 nor G-17 showed a significant association with inflammatory severity (Table 5).

**Table 5:** The relationship between biomarkers values and the severity of inflammation.

Inflammation	−	+	++	*p*
PG1(Mean ± SD)	145.67 ± 65.97	142.10 ± 57.49	116.66 ± 61.23	0.679
PG2 (Mean ± SD)	8.86 ± 6.14	6.81 ± 2.92	19.95 ± 17.96	**0**.002**
PG1/PG2 (Mean ± SD)	20.64 ± 9.12	22.01 ± 5.58	7.57 ± 5.29	**0**.008**
G-17 (Mean ± SD)	8.04 ± 12.78	5.38 ± 3.98	8.16 ± 7.93	0.628

−, no inflammation; +, mild inflammation; ++, moderate inflammation. SD, standard deviation.

Bold values indicate statistical significance (*p* < 0.05).

** *p* < 0.01.

## Discussion

CAG, a major sub-type of chronic gastritis, is characterized by the atrophy of the gastric mucosa. This condition involves the progressive loss of gastric glandular structures and may lead to intestinal metaplasia or pseudopyloric metaplasia (9, 10). Serological studies from various regions worldwide indicated that the prevalence of CAG can be as high as 27% in the general population (11, 12), while these percentage are highly dependent on the diagnostic criteria used (13). Given that the five-year progression rate from severe gastric mucosal atrophy to gastric cancer is approximately 6% (14), identifying individuals with CAG in the general population is clinically crucial.

Although gastroscopy is the unequivocal gold standard for CAG diagnosis and screening, primarily because it allows histological confirmation via pathological examination of tissue samples, its use for widespread screening is limited by high cost, patient discomfort, and poor compliance (15, 16). This underscores the critical need to develop and validate non-invasive screening methods. Such methods are desperately needed as triage tools to efficiently identify high-risk individuals for subsequent confirmatory gastroscopy, making population screening programs more efficient, cost-effective, and scalable.

Pepsinogens are the inactive precursors of pepsin, the primary enzyme responsible for protein digestion in the stomach. Under physiological conditions, PG1 is secreted exclusively by the oxyntic glands of the gastric corpus, whereas PG2 is produced by chief cells distributed throughout the garstric corpus, antrum, and duodenum. In gastric atrophy predominantly affecting the corpus-fundus region, serum levels of both PG1 and PG2 decrease. However, because the antrum is often spared, the decline in PG2 is generally less marked than that of PG1 (17). Consequently, serum pepsinogen levels might serve as a valuable indicator or gastric mucosal health and integrity. Gastrin, produced by G-cells in the gastric antrum (the lower part of the stomach), functions primarily to stimulate acid secretion from parietal cells in the gastric body. Whether serum biomarkers can aid in diagnosing CAG remains debated. Several studies support the use of pepsinogens and G-17 for diagnosing AG and for real-time monitoring of atrophic mucosa, particularly in autoimmune AG (13, 18, 19). In contrast, Cosmin et al. reported that the biomarker panel used in GastroPanel exhibited insufficient diagnostic accuracy for gastric atrophy in their cohort, with the PG1/PG2 ratio showing notably low reliability in cases of multifocal atrophy (6).

In this study, age was identified as a risk factor of AG. Serum levels of all evaluated biomarkers differed significantly between AG patients and non-AG participants. When sex and age were included as covariates in the ROC analysis, the biomarkers demonstrated diagnostic utility for AG, especially in female patients under 60 years old. No significant association was observed between biomarker levels and degree of AG severity in this study. A significant decrease in serum PG1 levels becomes apparent only when atrophy has reached a considerable severity, leading to a marked reduction in functional glandular cell. Due to its widespread production sources, PG2 levels usually decline less noticeably than PG1. Consequently, the PG1/PG2 ratio shows a significant decrease. This indicates that the gastric function panel hods good diagnostic value for detection severe atrophy, but has low sensitivity in distinguishing the degree of AG severity. Although several earlier studies have reported a link (5, 20, 21), the present analysis revealed no statistically significant correlation between *H. pylori* infection and CAG (*p* = 0.058). The relatively small sample size in this study is a potential reason for this lack of association.

The elevated PG1 in the AG group appears to contradict the classic view that atrophy reduces PG1. However, serum PG1 reflects the net balance between chief cell loss and inflammatory stimulation. Active inflammation, potentially driven by H. pylori, may stimulate residual chief cells to oversecrete PG1 and PG2, outweighing the effect of atrophy. Moreover, antral-predominant atrophy, likely common in our cohort, spares PG1-secreting cells in the corpus, allowing PG1 to remain elevated. In contrast, PG2 is more broadly secreted and inflammation-sensitive, explaining its greater elevation and the decreased PG1/PG2 ratio. Thus, in our population, inflammation may have a stronger net effect on pepsinogen levels than atrophy itself.

Several limitations should be acknowledged. The ROC cut-off values were determined based solely on our physical examination cohort using the Youden index, without internal validation such as bootstrap or cross-validation. Thus, the cut-off values may be overfitted and their generalizability remains to be verified. Future multicenter prospective studies with larger sample sizes are warranted for external validation. Specifically, the OLGA/OLGIM staging systems are the recommended international standards for gastric cancer risk stratification based on the extent and distribution of gastric atrophy and intestinal metaplasia. In the present study, the severity of gastric mucosal atrophy was assessed according to the Chinese Consensus on Chronic Gastritis (Chinese Society of Gastroenterology, 2006; updated in 2012 and 2017), which is the standard pathological grading system routinely used in clinical practice in China. We acknowledge that while this grading system reflects the local severity of atrophy, it does not incorporate the topographic distribution of atrophy across different gastric compartments (e.g., antrum, corpus, incisura angularis) as required by the OLGA/OLGIM systems. Therefore, it cannot fully replace OLGA/OLGIM staging for gastric cancer risk assessment. This is also a limitation of our study and future prospective studies with standardized biopsy protocols are warranted to apply OLGA/OLGIM staging for more comprehensive risk evaluation. Moreover, the number of patients with severe atrophy was extremely limited (*n* = 10), which substantially reduced the statistical power for subgroup comparisons according to atrophy grade. Therefore, the finding that no significant differences were observed across atrophy grades should be interpreted with caution. Finally, our cut-off values were established in a single-center setting without external validation, and were not compared with the widely used “gastrin trio” criteria. These issues should be addressed in future multi-center studies.

A precision screening approach that that utilizes the gastric function panel (PG1, PG2, PG1/PG2 ratio, and G-17) within defined age/sex strata demonstrates significant value for detecting AG. This methodology provides a non-invasive and cost-effective solution for initial risk assessment. Its primary clinical utility lies in its ability to identify high-risk individuals with greater accuracy, which subsequently refines the selection of candidates for confirmatory endoscopy. This not only enhances the feasibility of large-scale screening programs but also ensures the efficient allocation of endoscopic resources.

## Conclusion

Serum gastric function biomarkers could serve as diagnostic tools for AG, particularly among female patients under 60 years old. No significant association was found between these biomarkers and atrophy severity, likely due to the limited sample. PG1 and the PG1/PG2 ratio were significantly associated with the severity of inflammation.

## Data Availability

The data analyzed in this study is subject to the following licenses/restrictions: The datasets generated during this study are available from the corresponding author on reasonable request. Requests to access these datasets should be directed to Wei Han, hanwei@sdu.edu.cn.

## Supplementary material

Supplementary Figure S1 ROC curves of PG1, PG2, PG1/PG2 ratio, and G-17 for the diagnosis of AG in Hp-positive patients, stratified by sex and age subgroups.

Supplementary Figure S1 ROC curves of PG1, PG2, PG1/PG2 ratio, and G-17 for the diagnosis of AG in Hp-negative patients, stratified by sex and age subgroups.
